# Clopidogrel-induced non-diabetic hypoglycemia reported from Tikur Anbessa Specialized Hospital, Addis Ababa, Ethiopia: a case report

**DOI:** 10.1186/s13256-023-04166-8

**Published:** 2023-11-05

**Authors:** Kibret Enyew Belay, Saba Belay Urge, Yidnekachew Asrat Birhan, Paulos Efrem, Theodros Aberra Alemneh

**Affiliations:** 1 Department of Internal Medicine, Endocrinology and Metabolism Unit, Addis Ababa University, Addis Ababa, Ethiopia; 2 Department of Internal Medicine, Cardiology Unit, Addis Ababa University, Addis Ababa, Ethiopia

**Keywords:** Clopidogrel, Non-diabetic hypoglycemia

## Abstract

**Background:**

Recurrent episodes of hypoglycemia may be caused by several factors, including drugs, critical illnesses, hormonal deficiency, non-islet cell tumor endogenous hyperinsulinism, and accidental, surreptitious, or malicious hypoglycemia. Multiple drugs have been previously reported as causes of hypoglycemia, with moderate and low-quality evidence. However, Clopidogrel as a cause of non-diabetic hypoglycemia is rarely reported. Here we describe a single non-diabetic patient who experienced recurrent episodes of hypoglycemia after initiation of clopidogrel for clinical suspicion of acute coronary syndrome.

**Case presentation:**

The patient, a 33-year-old Ethiopian male with documented hypertension on antihypertensive medication, has started receiving treatment for acute coronary syndrome after experiencing angina symptoms. He experienced hypoglycemia following the start of Clopidogrel, but it subsided once it was stopped. Currently, he has a follow-up at the cardiac clinic with a normal measurement of his serum blood glucose level.

**Conclusion:**

Non-diabetic hypoglycemia is a rare illness characterized by low blood glucose levels in people who do not have diabetes. Patients with severe hypoglycemia may become unconscious or have seizures as a result of low blood sugar. Severe hypoglycemia is fatal and must be treated as soon as possible. Therefore, if non-diabetic hypoglycemia occurs, a thorough evaluation of the causes is essential, particularly any potential drug as a cause of hypoglycemia should be evaluated.

## Introduction

Hypoglycemia is defined by Whipple’s triad, which manifests as the development of autonomic or neuroglycopenic symptoms (palpitations, sweating, anxiety, hunger, or confusion); a low plasma glucose (PG) level below 55 mg/dl; and symptoms responding to the administration of carbohydrate [1, 2]. Hypoglycemia is a rare occurrence in individuals without diabetes but is common in sulfonylureas, glinides, or insulin-treated diabetes [3].

According to a large study done in 2012, 71 hypoglycemic episodes were identified among the 37,898 admissions at a cut-off of 60 mg/dl and an estimated 50 episodes at a cut-off of 54 mg/dl per 10,000 admissions [4]. In the absence of diabetes, hypoglycemia in ill or medicated individuals can be caused by many drugs, critical illnesses, endocrine deficiencies, or non-islet cell tumors; in seemingly healthy individuals, it may be caused by endogenous hyperinsulinism or by various accidental, surreptitious, or malicious mechanisms [5]. A Systematic Review of 448 eligible studies done on drug-Induced Hypoglycemia showed that only seven drugs (Cibenzoline, Clinafloxacin, Gatifloxacin, Glucagon, Indomethacin, Pentamidine, and Quinine) support an association with hypoglycemia with moderate-quality evidence [6].

Clopidogrel-induced hypoglycemia is a rare disease [7–10]. There are only a few case reports that showed an association between Clopidogrel and nondiabetic hypoglycemia [9, 11, 12]. The objective of this study is to strengthen the association of Clopidogrel with hypoglycemia in non-diabetic patients. A study on a 79-year-old white male showed that hypoglycemia occurred after 3 weeks of clopidogrel initiation. Further evaluations confirm the presence of anti-insulin antibodies as a cause of Clopidogrel-Induced Insulin Autoimmune Syndrome [8]. Hypoglycemia can be the primary symptom of the insulin autoimmune syndrome (IAS), which is brought on by the sulfhydryl group of the clopidogrel metabolite. The hepatic enzymes CYP2C19, CYP2B6, and CYP3A4 convert clopidogrel into a sulfhydryl derivative after consumption, further damaging the disulfide bonds of the insulin molecule. This causes immunogenicity and promotes the growth of T cells as well as the creation of autoimmune antibodies against insulin. These antibodies will then attach to insulin and dissociate in an uncontrolled manner [9].

Populations with the genetic background of the HLA-DRB1*0406, DRB1*0403, and DQB1*0302 sensitive alleles experience increased incidence [3]. Asian participants and aging population were shown to be risk factors for clopidogrel-associated hypoglycemia [9]. Hypoglycemia has also been reported in diabetic patients when it is added on top of sulfonylureas [13].

## Case summary

A 33-year-old Ethiopian male patient with a history of hypertension and hypertensive heart disease for the past 6 years but on treatment for 3 years on enalapril (5 mg po daily), bisoprolol (5 mg po daily), and aspirin (81 mg po daily).

He currently presents with a squeezing type of retrosternal chest pain, diaphoresis, and vomiting of ingested matter of 3 days duration, for which he was admitted to Gondar Hospital for 3 weeks and treated for possible acute coronary syndrome with dual antiplatelet therapy (aspirin and clopidogrel), and atorvastatin 80 mg po daily. However, there was no evidence of myocardial injury or ischemia on troponin, electrocardiogram, or echocardiography. After admission to the intensive care unit, he started to develop recurrent symptomatic hypoglycemia, with the lowest record being 21 mg/dl, which improves with eating. He was taking meals every 3 hour due to the symptoms. The measurements were confirmed in the hospital laboratory in addition to a glucometer-based investigation.

He has no personal or family history of diabetes mellitus, dyslipidemia, smoking, substance abuse, a history of change in mentation, or a family history of cardiac illness. All his family members are healthy, and they are not taking any drugs. The patient's hypoglycemia was approached systematically based on guidelines recommendations [5]. There were no clinical or laboratory suggestions of hormonal deficiencies, critical illnesses, non-islet cell tumors, or others as causes of hypoglycemia. Among the drugs he was taking, bisoprolol was discontinued and shifted to diltiazem. However, his blood sugar was persistently low. On subsequent follow-up, clopidogrel was discontinued, and his blood glucose normalized since the next day of clopidogrel withdrawal. He has repeated measurements of blood sugar levels above 70 mg/dl.

There were no pertinent physical findings, including cardiac and chest evaluations. Basic laboratory tests, including complete blood count, renal function test, and serum electrolytes, were all normal. He also has normal HbA1C (4.8%), normal serum cortisol (18.9 mcg/dl), non-revealing urine analysis, normal thyroid function tests (Free T4 1.56 ng/dl, TSH 2.4 mIU/L), a normal coagulation profile (INR = 0.89, PT = 12.8 seconds, aPTT = 28.5 seconds), and normal 24-hour urine catecholamines (epinephrine 4.68 mcg, norepinephrine 28.56 mcg, dopamine 225.4 mcg, normetanephrine 380 mcg, metanephrines 80.46 mcg).

He has a normal resting sinus rhythm, but stress electrocardiography showed intermediate risk; he had exercise-limiting anginal pain before he achieved his estimated heart rate, and the exercise test was discontinued. Echocardiography showed normal 2D and Doppler imaging and no evidence of resting ischemia. The angiography finding was normal epicardial coronary arteries.

## Discussion

Hypoglycemia is an uncommon clinical problem in individuals without diabetes mellitus [14]. It can occur in the fasting or postprandial state, and some individuals experience both fasting and postprandial hypoglycemia [15]. Fulfillment of Whipple's triad supports the presence of pathologic rather than physiologic hypoglycemia [5].

For individuals with evidence of a hypoglycemic disorder (fulfillment of Whipple's triad or high clinical suspicion based on home glucose monitoring), a thorough clinical history and physical examination can help identify the most likely cause(s) of the hypoglycemia and inform the next steps in evaluation [16, 17].

To our knowledge, this is a rare case report of hypoglycemia in a patient following clopidogrel exposure. In our patient, Whipple’s triad of symptomatic hypoglycemia (plasma glucose level less than 55) and resolution following meal were documented before the initiation of medical evaluation [5]. Clopidogrel's identification as the cause of episodes of hypoglycemia is highly supported by the temporal relationship between those two events. This case is also unique as it happens after initiating clopidogrel for possible acute coronary syndrome by only clinical assumption which was finally found to have normal angiography.

The pathophysiological explanation may be due to the development of insulin-autoimmune syndrome after exposure to clopidogrel [7]. The antibody test was not done due to the patient's financial problems and the improvement of the symptoms, which is the limitation of this study. The acute management of the patient is similar to other causes of hypoglycemia and the discontinuation of the offending drug. The patient is currently on follow-up at the cardiac clinic with no record of hypoglycemia for the past 2 months.

## Conclusion

Although hypoglycemia is uncommon in people without diabetes, a comprehensive evaluation and diagnosis is required because the situation is a medical emergency. Drugs are the number one factor contributing to non-diabetic hypoglycemia. As a result, each drug a patient is taking needs to be adequately evaluated.

## Data Availability

All data sets on which the conclusions of the case report are based are to be available as a medical record document and available from the corresponding author on reasonable request from the editors.

## Abbreviations

HbA1C: Hemoglobin A1C
T4: Thyroxine
TSH: Thyroid stimulating hormone
INR: International normalized ratio
PT: Prothrombin time
aPTT: Activated partial thromboplastin time
